# Neutrophil-Lymphocyte Ratio as an Initial Screening Biomarker for Differential Diagnosis of Cushing's Syndrome from Nonfunctional Adenoma in Patients with an Adrenal Mass

**DOI:** 10.1155/2021/6635594

**Published:** 2021-02-15

**Authors:** Wei Wang, Jianing Wang, Cheng Shen, Sainan Zhu, Ying Gao, Junqing Zhang

**Affiliations:** ^1^Department of Endocrinology, Peking University First Hospital, No. 8 Xishiku Street, Beijing 100034, China; ^2^Department of Endocrinology, Beijing Longfu Hospital, No. 18 Art Museum East Street, Beijing 100010, China; ^3^Department of Urology, Peking University First Hospital, No. 8 Xishiku Street, Beijing 100034, China; ^4^Department of Medical Statistics, Peking University First Hospital, No. 8 Xishiku Street, Beijing 100034, China

## Abstract

**Objective:**

Assessing excess adrenal hormones is important in patients with adrenal mass. Current screening tests for excess cortisol hormones are complex, so it cannot be done sometimes due to the limited medical resources. The aim of the study was to evaluate whether the neutrophil-lymphocyte ratio (NLR) can be used as an initial screening biomarker for Cushing's syndrome (CS) in patients with an adrenal mass.

**Methods:**

This retrospective study included a total of 185 patients with CS and 185 patients with nonfunctional adrenal adenoma (matched 1 : 1 by sex, body mass index, and discharge date). The NLR was compared between the two groups. The association between NLR and serum and urinary cortisol concentrations was analyzed, and an NLR cut-off value for CS screening was calculated.

**Results:**

NLR (3.38 (2.33, 5.45) vs. 2.13 (1.74, 3.00), *P* < 0.001) was significantly higher in the CS group than in the nonfunctional adenoma group. In CS patients, the NLR was positively associated with serum cortisol concentrations at 8 am, with 24-hour urine free cortisol and with serum cortisol after a 1 mg dexamethasone suppression test (*P* < 0.001 each). An NLR cut-off of 2.2 had a sensitivity of 80.0% and a specificity of 54.05%. The weighted Youden index for the NLR was similar to that of the 24-hour urine free cortisol and late-night serum cortisol tests, which are recommended initial tests for CS diagnosis.

**Conclusion:**

The NLR may be useful for initial screening for CS among patients with an adrenal mass as an easy and convenient marker.

## 1. Introduction

The frequency of detection of adrenal masses has increased in recent years after the introduction of high-resolution cross-sectional imaging. Previous studies report a frequency of around 3% in subjects aged 50 years, increasing to about 10% in elderly individuals [1–5]. The European Society of Endocrinology and European Network for the Study of Adrenal Tumors guidelines on adrenal incidentaloma recommend that adrenal hormone concentrations in patients with adrenal incidentaloma are measured to identify those with adrenal hormone excess [6]. About 12% of patients with adrenal incidentaloma have Cushing's syndrome (CS), a condition characterized by constitutive cortisol-secreting adenomas and associated with severe morbidity and elevated mortality. Despite available treatments for comorbidities, patients with active CS continue to have a standardized mortality rate 1.7- to 4.8-fold greater than the general population [7–11]. Early diagnosis of CS can improve patient prognosis.

The Chinese experts' consensus for Cushing's syndrome (2011) recommends two steps for CS diagnosis. Initial screening includes measurement of 24-hour urine free cortisol (UFC), late-night saliva cortisol, and serum cortisol rhythms. If the results in initial screening are abnormal, further diagnostic indicators include serum cortisol levels greater than 1.8 *μ*g/dL after overnight 1 mg dexamethasone or low-dose dexamethasone (2 mg/day for 48 hours) suppression tests (LDDST), [12]. Since tests for serum cortisol and UFC are available only in some large hospitals and the saliva cortisol test can be performed by only a few hospitals, special endocrinology tests may not always be available in underdeveloped regions of China. Initial screening and further diagnostic tests for CS cannot be performed due to stringent requirements regarding detection methods and sample collection; this is a problem for primary care clinics or underdeveloped regions where medical resources are scarce. Therefore, more easily measurable biomarkers are needed to screen for CS.

Previous studies show that elevated white blood cell (WBC) counts in Cushing's disease are associated with hypercortisolism; this is because glucocorticoid receptors expressed by WBCs play a role in cell adhesion and WBC recruitment from the bone marrow [13]. Additionally, there is a significant positive correlation between decreased UFC levels and decreased WBC counts after surgery; similar changes in WBC counts are observed in pediatric CS patients [14]. Furthermore, neutrophil counts are significantly higher, and lymphocyte counts are significantly lower, in CS patients than in controls. The neutrophil-lymphocyte ratio (NLR) is an index calculated by dividing the neutrophil count by the lymphocyte count, which may be associated with cortisol levels. Studies show that the NLR is associated with disease activity and prognosis in patients with tumors, cardiovascular disease, and autoimmune disease [15–19]. However, little is known about the NLR in patients with CS. The NLR is easier to measure during routine blood testing than during current initial tests for CS. The aim of the present study was to investigate whether the NLR is an appropriate screening biomarker for CS.

## 2. Materials and Methods

### 2.1. Study Population

The electronic medical record system of Peking University First Hospital was searched to identify patients with an adrenal mass confirmed by CT scanning from January 2014 to March 2019. Adrenal CS was diagnosed as serum cortisol levels greater than 1.8 *μ*g/dL after LDDST, according to the criteria of the Endocrine Society clinical practice guidelines [20]. Nonfunctional adrenal adenoma was diagnosed by functional assessment, excluding patients with CS [20], primary aldosteronism [21], pheochromocytoma [22], and infiltrative diseases such as adrenal lymphoma and metastatic carcinoma. Patients were included if they had been hospitalized for an adrenal mass, their medical data were complete, and they were discharged with a diagnosis of adrenal CS or nonfunctional adrenal adenoma. Patients were excluded if they had an active infection, a disease of the hematologic system, cardiovascular disease, autoimmune disease, or a nonadrenal tumor. Pregnant women were also excluded, as were those with factors that could affect neutrophil or lymphocyte counts. The diagnostic criteria for an active infection were symptoms or signs of infection, or detection of infection in samples of urine, stool, or other body fluids, or use of antibiotics at the time.

### 2.2. Ethics

This study conformed to the principles of the Declaration of Helsinki. The study protocol was approved by the Ethics Committee of Peking University First Hospital (approval number 2019-300).

### 2.3. Study Design

This retrospective analysis included patients treated at a single research hospital. Factors recorded included demographic characteristics (e.g., age, sex, height, weight, and body mass index (BMI)), history of illness, diagnosis at discharge, results of physical examination (e.g., blood pressure and heart rate on the first day of hospitalization), routine blood tests, and measurements of plasma glucose, lipid, electrolyte, and C-reactive protein concentrations (CRP). Other factors included serum cortisol and ACTH concentration rhythms, 24-hour UFC, 8 am serum cortisol after 1 mg DST, the results of adrenal CT scans, and pos-operative histopathology. All patients were admitted to the hospital for a period of 48 hours or longer before midnight cortisol sampling to avoid false-positive responses due to the stress of hospitalization. The patient was in sleeping before midnight sampling; the blood sample must be drawn within 5–10 minutes of waking [20]. Patients with CS were divided into those with overt CS and those with adrenal incidentaloma with mild autonomous cortisol excess (AIMCE), depending on whether they presented with or without specific signs and symptoms of CS [23, 24]. AIMCE was defined as autonomous cortisol secretion in the absence of the classical clinical features of Cushing's syndrome in the adrenal incidentaloma [6].

Histopathology of ACTH-independent CS usually includes adrenocortical adenoma, adrenocortical hyperplasia, oncocytic adenoma, and adrenocortical carcinoma. NLR was calculated by dividing the neutrophil count (×10^9^/L) by the lymphocyte count (×10^9^/L). The weighted Youden index was calculated as 2 × [*ω* × sensitivity + (1 − *ω*) × specificity] − 1, where *ω* is the weighted coefficient of sensitivity. For the screening test, sensitivity carried more weight, so *ω* was set at 0.7.

### 2.4. Statistical Analysis

A normality test identified age, BMI, blood pressure, plasma potassium, plasma lipid, neutrophil count, and lymphocyte count as being normally distributed, whereas the NLR, serum cortisol and ACTH concentrations, CRP levels, the longest diameter of adrenal nodules, HbA1c, and fasting plasma glucose were nonnormally distributed. Normally distributed parameters were expressed as the mean ± standard deviation and were analyzed using Student's *t* tests. Nonnormally distributed parameters were expressed as the median (interquartile range) and analyzed using the Mann–Whitney *U* test. The NLR of multiple subgroups was compared using the Kruskal-Wallis test. Categorical data were reported as percentages and compared using the Chi-square test. The correlation between NLR and serum and urinary cortisol levels was determined by Spearman's correlation analysis. The cut-off NLR value for CS was determined by receiver operating characteristic (ROC) curves analysis. Areas under the ROC curves for the NLR, neutrophil count, and lymphocyte count were compared using MedCalc software. The sensitivity and specificity of the different tests were compared using McNemar's test. A *P* value < 0.05 was considered statistically significant.

## 3. Results

### 3.1. Characteristics of the CS and Nonfunctional Adrenal Mass Groups

A review of medical records identified 185 patients who had been diagnosed with adrenal CS during the study period. These patients were matched 1 : 1 by sex, BMI (±0.5 kg/m^2^), and discharge date, with 185 patients diagnosed with a nonfunctional adrenal mass. In addition to serum cortisol and ACTH concentrations, there were significant between-group differences in UFC, age, systolic and diastolic blood pressure, total cholesterol, plasma potassium concentrations, CRP levels, and adrenal nodule size (Table 1).

### 3.2. The NLR in the CS and Nonfunctional Adrenal Mass Groups

**Figure 1:**
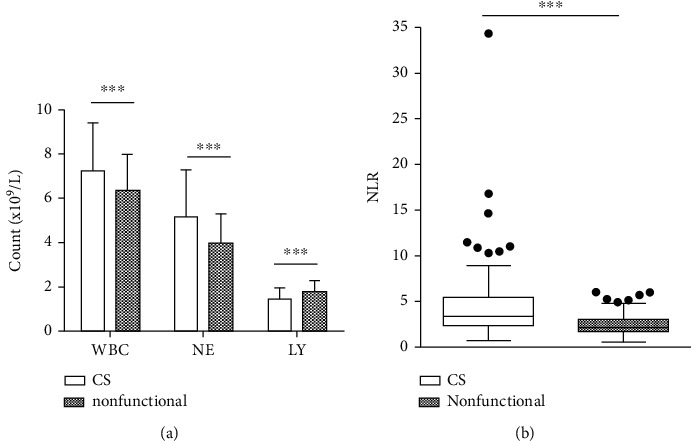
NLR and white blood cell, neutrophil, and lymphocyte counts in groups of patients with CS or a nonfunctional adrenal mass. (a) Neutrophil and lymphocyte counts in the two groups. The mean white blood cell counts (7.25 ± 2.16 vs. 6.35 ± 1.63 (×10^9^/L), *P* < 0.001) and neutrophil count (5.17 ± 2.11 vs. 3.96 ± 1.34 (×10^9^/L), *P* < 0.001) were significantly higher, and the mean lymphocyte count was significantly lower (1.45 ± 0.53 vs. 1.75 ± 0.53 (×10^9^/L), *P* < 0.001], in the CS group. Data are presented as the mean ± SD. ^∗∗∗^*P* < 0.001 (Student's *t* test). (b) NLR for the two groups. The NLR (3.38 (2.33, 5.45) vs. 2.13 (1.74, 3.00), *P* < 0.001) was significantly higher in the CS group. Data are presented as the median (interquartile range). ^∗∗∗^*P* < 0.001 (Mann–Whitney tests). The number in each group = 185. Abbreviations: WBC: white blood cell count; NE: neutrophil count; LY: lymphocyte count; NLR: neutrophil-lymphocyte ratio; CS: Cushing's syndrome.

WBC counts (7.25 ± 2.16 vs. 6.35 ± 1.63 (×10^9^/L), *P* < 0.001) and neutrophil counts (5.17 ± 2.11 vs. 3.96 ± 1.34 (×10^9^/L), *P* < 0.001) were significantly higher, and lymphocyte counts (1.45 ± 0.53 vs. 1.75 ± 0.53 (×10^9^/L), *P* < 0.001) were significantly lower in the CS group than in patients with a nonfunctional adrenal mass. The NLR (3.38 (2.33, 5.45) vs. 2.13 (1.74, 3.00), *P* < 0.001) was significantly higher in the CS group than in the nonfunctional adrenal mass group (Figure 1).

### 3.3. Correlation between Serum and Urinary Cortisol Levels and the NLR in the CS Group

**Figure 2:**
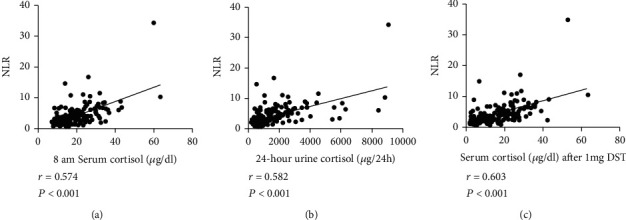
Scatter plots showing the correlation between the NLR and cortisol levels in the CS group. (a) NLR vs. 8 am serum cortisol concentration. (b). NLR vs. 24-hour urinary cortisol. (c) NLR vs. 8 am serum cortisol after 1 mg DST. *R* and *P* values were determined by Spearman's correlation analysis. *N* = 185. Abbreviations: NLR: neutrophil-lymphocyte ratio; CS: Cushing's syndrome; DST: dexamethasone suppression test.

After adjusting for BMI, the NLR in the CS group correlated significantly with 8 am serum cortisol (*r* = 0.574, *P* < 0.001), 24-hour UFC (*r* = 0.582, *P* < 0.001), and 8 am serum cortisol levels after the 1 mg DST (*r* = 0.603, *P* < 0.001) (Figures 2(a)–2(c)). These positive correlations were also observed after removing an outlier (*r* was 0.567 (NLR vs. 8 am serum cortisol concentration), 0.575 (NLR vs. 24-hour UFC), and 0.596 (NLR vs. 8 am serum cortisol after the 1 mg DST); *P* < 0.001 for each; data not shown).

### 3.4. Comparison of the NLR in Different CS Subgroups

A comparison of the NLR between the overt CS and AIMCE subgroups showed that the NLR was significantly higher in patients with overt CS than in those with AIMCE (Table 2). Next, the NLR was compared between different histopathologic subgroups. Histopathologic analysis of the 133 patients with ACTH-independent CS who underwent surgery resulted in a diagnosis of adrenocortical adenoma in 82 patients, adrenocortical hyperplasia in 11, oncocytic adenoma in 35, and adrenocortical carcinoma in five. The NLR in these subgroups was 4.04 (2.80, 6.15), 3.40 (2.73, 5.81), 4.18 (2.18, 6.21), and 3.43 (2.74, 5.23), respectively, and did not differ significantly among them.

### 3.5. Area under the ROC Curves for the NLR for Screening Patients with an Adrenal Mass for CS and Calculating the NLR Cut-Off Value

**Figure 3:**
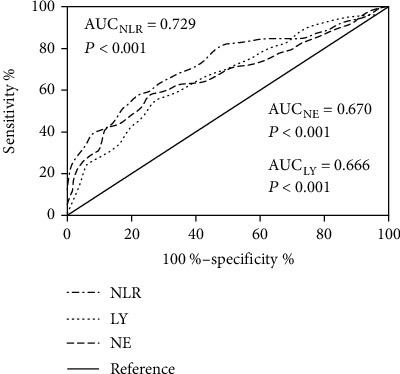
Receiver operating characteristic curves of the NLR, NE, and LY for diagnosis of CS. *N* = 370. Abbreviations: NLR: neutrophil-lymphocyte ratio; NE: neutrophil count; LY: lymphocyte count; AUC: area under the curve; CS: Cushing's syndrome.

The 370 samples from the two groups were combined to assess the cut-off value for the NLR when screening for CS. ROC analysis showed that the area under the curve for the NLR was 0.729 (*P* < 0.001). The area under the ROC curve for NLR was significantly higher than that for the neutrophil count (*P* = 0.0043) and that for the lymphocyte count (*P* = 0.0067) (Figure 3). A cut-off NLR value of 2.2 for CS diagnosis yielded a sensitivity of 80.0% and a specificity of 54.05%.

### 3.6. Comparison of the NLR with Other Markers for Screening CS

Finally, we compared the sensitivity and specificity of the NLR with those of 24-hour UFC (cut-off, upper limit of normal range), late-night serum cortisol (cut-off, 1.8 *μ*g/dL), and 1 mg overnight DST (cut-off, 1.8 *μ*g/dL). The sensitivity and specificity of the NLR was between those of the 24-hour UFC and those of the late-night serum cortisol tests. The weighted Youden index of these biomarkers was calculated to show diagnostic accuracy (Table 3). The diagnostic accuracy of the NLR was similar to that of the 24-hour UFC and the late-night serum cortisol tests.

## 4. Discussion

Previous studies demonstrate that elevated WBC counts in CS are associated with hypercortisolism. Moreover, lymphocyte counts are significantly lower and neutrophil counts significantly higher, in CS patients than in controls. After surgery, WBC counts decrease with the decline in serum cortisol [13, 14]. We also observed these changes in the CS patients in our study. Additionally, many studies report that the mechanism of changes in lymphocyte counts and neutrophil counts involves an increase in cortisol concentration. The bone marrow of CS patients release high numbers of neutrophils. The marginated pool of neutrophils is mobilized into the circulating pool, due to elevation of cortisol levels. Meanwhile, cortisol inhibits apoptosis of human neutrophils and increases the half-life of circulating neutrophils. At the same time, hypercortisolemia reduces lymphocyte numbers via an immunosuppressive mechanism [13, 14, 25–28]. However, tests based solely on the results of data obtained from routine blood tests are insufficient for screening patients with suspected CS. Because the NLR amplified the difference than solely blood cell counts, it can distinguish between the two groups much better than blood cell counts. In our study, we found that the NLR was significantly higher in patients with CS than in those with a nonfunctional adrenal mass, and subgroup analysis revealed that the NLR was significantly higher in patients with overt CS than in those with AIMCE. The NLR was positively associated with cortisol levels in CS patients. These findings suggest that the NLR may reflect, at least in part, cortisol concentrations and may be a potential biomarker in initial CS tests.

Previous studies report that the NLR is valuable for predicting disease activity and prognosis in patients with tumors, cardiovascular disease, and autoimmune disease [15–19]. A higher NLR is associated with autoimmune disease activity and with poorer prognosis in patients with tumors and cardiovascular disease. To date, no published studies have assessed the relationships between the NLR and a diagnosis of CS. Here, we found that the NLR in patients with CS was significantly higher than that in individuals with a nonfunctional adrenal mass. Conversely, the CRP concentration was lower in CS patients than in the nonfunctional adrenal mass group, indicating that differences in the NLR between these two groups do not reflect inflammatory status.

We found that the CS group was significantly younger than the nonfunctional group. A previous study shows that the NLR is positively associated with age; older people have a relatively high NLR [29]. This suggests that the higher NLR in the CS group was not due to age differences. Because patients with CS show varied clinical complications [30], an adrenal mass might be detected earlier than in patients with asymptomatic nonfunctional adenoma. In our study, we excluded those with adrenal lymphoma and adrenal metastatic carcinoma. In these cases, the NLR does not reflect cortisol levels; this is because adrenal malignant masses might affect inflammatory parameters, including the NLR.

Current screening tests for CS include late-night saliva/serum cortisol concentration, 24-hour UFC, and DSTs [20]. Although these tests have high diagnostic accuracy and are feasible, their widespread use is limited by stringent requirements with respect to specimen collection and patient compliance, as well as by the need for specialized equipment, which is a problem in underdeveloped countries and small hospitals. By contrast, the NLR can be calculated easily from routine blood test data. Compared with traditional initial tests for CS diagnosis, the NLR has several advantages. In the case of the 24-hour UFC test, it is not easy for patients to provide a complete set of 24-hour urine samples of the appropriate total volume. Moreover, excessive fluid intake and other physiological or pathological conditions can affect UFC test results [31]. A falsely low UFC can occur when the creatinine clearance rate falls below 60 mL/min [32]. In the case of the late-night saliva/serum cortisol test, samples have to be collected late at night, which is inconvenient. Also, it cannot be used for shift workers or for those with variable bedtimes [20]. Patients who smoke cigarettes have higher salivary cortisol concentrations than nonsmokers [33]. Pain and stress caused by blood drawing can affect serum cortisol levels. Studies show that the sensitivity and specificity of UFC for CS diagnosis are 65–97% and 56–100%, respectively, due to the use of different cut-off values and methods [34, 35]. The sensitivity and specificity of the late-night serum cortisol test with a cut-off of 1.8 *μ*g/dL are 100% and 20.2%, respectively [36], whereas those of postdexamethasone serum cortisol tests to less than 1.8 *μ*g/dL are greater than 95% and 80%, respectively [37]. The weighted Youden index suggests that the diagnostic accuracy of the NLR in our study is similar to that of the late-night serum cortisol concentration and 24-hour UFC tests.

Thus, we propose that NLR may be an initial screening test for patients with an adrenal mass that lack factors that could affect neutrophil or lymphocyte counts. Calculating the NLR is easy and can be done in primary care clinics and healthcare facilities in underdeveloped regions. For attention, although WBC counts, neutrophil counts, and the NLR were significantly higher in the CS group than in the nonfunctional group, WBC counts and neutrophil counts are still in the normal range.

Taken together, the results presented herein indicate that the NLR is an easily measurable, reproducible, and inexpensive marker for initial screening of CS in patients with an adrenal mass. Undoubtedly, all patients with an adrenal mass require evaluation in an endocrinological unit. A complete adrenal function evaluation, including cortisol, aldosterone, and catecholamine levels, is required. Also, a 1 mg overnight DST is the most important parameter for diagnosis under the current guidelines, and this can be done easily in endocrinological units. The NLR test, which can be performed in nonspecialist units, could also provide useful information when no endocrinologist is available.

The present study has several limitations. First, it was performed at a single center in China and included a relatively small number of patients. Second, the retrospective design means that the NLR could only be measured preoperatively. It is unclear whether the NLR in patients with CS falls after adrenalectomy, or whether changes in the NLR predict CS outcome. Further studies are needed to confirm our results.

## 5. Conclusions

The NLR is easier to calculate during routine blood testing than current initial tests for CS. We found that the NLR in patients with CS was significantly higher than that in patients with a nonfunctional adrenal mass. A high NLR correlated positively with cortisol levels. A NLR ≥ 2.2 may be used as a cut-off for screening tests for initial diagnosis of CS in patients with an adrenal mass.

## Figures and Tables

**Table 1:** Baseline characteristics of the CS and nonfunctional adrenal mass groups.

	CS (*n* = 185)	Nonfunctional (*n* = 185)	*P* value
Male (%)	61 (32.97%)	61 (32.97%)	—
Age (years)	47.68 ± 13.82	54.71 ± 10.89	<0.001
BMI (kg/m^2^)	25.98 ± 3.75	26.00 ± 3.66	0.947
Hypertension (%)	151 (81.62%)	124 (67.03%)	0.001
Diabetes (%)	63 (34.05%)	51 (27.57%)	0.177
SBP (mmHg)	144.65 ± 18.84	138.77 ± 18.30	0.002
DBP (mmHg)	88.17 ± 14.60	80.44 ± 12.79	<0.001
HbA1c (%)	6.0 (5.7, 6.8)	6.0 (5.7, 6.6)	0.573
FPG (mmol/L)	5.31 (4.72, 6.34)	5.47 (5.04, 6.30)	0.141
Plasma K+ (mmol/L)	3.55 ± 0.44	3.70 ± 0.33	<0.001
TG (mmol/L)	1.64 ± 0.80	1.75 ± 1.105	0.315
TCHO (mmol/L)	4.97 ± 1.16	4.55 ± 0.97	<0.001
8 am serum cortisol (*μ*g/dL)	16.92 (12.32, 23.99)	12.35 (9.71, 16.87)	<0.001
4 pm serum cortisol (*μ*g/dL)	13.61 (8.87, 21.52)	6.66 (5.11, 9.62)	<0.001
0 am serum cortisol (*μ*g/dL)	11.67 (6.33, 19.35)	2.67 (1.77, 4.50)	<0.001
8 am ACTH (pg/mL)	2.00 (0.99, 7.74)	18.54 (12.30, 26.62)	<0.001
4 pm ACTH (pg/mL)	1.57 (0.99, 4.47)	10.72 (7.51, 15.96)	<0.001
0 am ACTH (pg/mL)	1.30 (0.99, 2.93)	6.99 (3.97, 11.72)	<0.001
Urine cortisol (*μ*g/24 h)	963.3 (568.1, 1850.0)	539.8 (412.2, 717.9)	<0.001
CRP (mg/L)	0.79 (0.30, 1.89)	1.24 (0.65, 2.33)	0.001
Longest diameter of adrenal nodule (cm)	2.70 (2.20, 3.60)	1.60 (1.00, 2.30)	<0.001

Results are presented as the mean ± SD, median (interquartile range), or *n* (%). Abbreviations: CS: Cushing's syndrome; BMI: body mass index; SBP: systolic blood pressure; DBP: diastolic blood pressure; FPG: fasting plasma glucose; TG: triglyceride; TCHO: total cholesterol; ACTH: corticotropin; CRP: C-reactive protein.

**Table 2:** Comparison of hormone concentrations, NLR, and PLR in patients with overt CS and AIMCE.

	Overt CS (*n* = 141)	AIMCE (*n* = 44)	*P*
8 am serum cortisol (*μ*g/dL)	19.76 (13.79, 25.45)	13.82 (10.51, 16.06)	<0.001
8 am ACTH (pg/mL)	1.23 (0.90, 4.32)	8.43 (4.30, 11.85)	<0.001
NE (×10^9^/L)	5.58 ± 2.16	3.85 ± 1.22	<0.001
LY (×10^9^/L)	1.35 ± 0.47	1.77 ± 0.58	<0.001
NLR	4.00 (2.70, 6.18)	2.16 (1.60, 3.07)	<0.001

Results are presented as the mean ± SD or as the median (interquartile range). Abbreviations: ACTH: corticotropin; NE: neutrophil count; LY: lymphocyte count; NLR: neutrophil-lymphocyte ratio; CS: Cushing's syndrome; AIMCE: adrenal incidentaloma with mild autonomous cortisol excess.

**Table 3:** Comparison of different initial screen markers used to diagnose CS.

	Sensitivity	Specificity	Weighted Youden index^△^
0 am serum cortisol (*μ*g/dL)	98.8%^∗∗∗^	26.6%^∗∗∗^	0.543
24-hour urine free cortisol	67.1%^∗∗^	70.5%^∗∗^	0.362
Serum cortisol after 1 mg DST (*μ*g/dL)	100%^∗∗∗^	89.1%^∗∗∗^	0.935
NLR	80.0%	54.1%	0.445

Abbreviations: DST: dexamethasone suppression test; NLR: neutrophil-lymphocyte ratio; CS: Cushing's syndrome. ^∗∗^*P* < 0.01 and ^∗∗∗^*P* < 0.001, *vs.* NLR.

^△^weighted coefficient of sensitivity *ω* = 0.7.

## Data Availability

The datasets used and analyzed during the current study are available from the corresponding author on reasonable request.
